# Jumping with adhesion: landing surface incline alters impact force and body kinematics in crested geckos

**DOI:** 10.1038/s41598-021-02033-4

**Published:** 2021-11-29

**Authors:** Timothy E. Higham, Mara N. S. Hofmann, Michelle Modert, Marc Thielen, Thomas Speck

**Affiliations:** 1 Department of Evolution, Ecology, and Organismal Biology, University of California, Riverside, CA 92521 USA; 2 Plant Biomechanics Group & Botanic Garden, University of Freiburg, Freiburg, Germany; 3 FIT, Freiburg Center for Interactive Materials and Bioinspired Technologies, Freiburg, Germany; 4 FMF, Freiburg Materials Research Center, Freiburg, Germany; 5 Cluster of Excellence livMatS@FIT, Freiburg, Germany

**Keywords:** Animal behaviour, Biomechanics

## Abstract

Arboreal habitats are characterized by a complex three-dimensional array of branches that vary in numerous characteristics, including incline, compliance, roughness, and diameter. Gaps must often be crossed, and this is frequently accomplished by leaping. Geckos bearing an adhesive system often jump in arboreal habitats, although few studies have examined their jumping biomechanics. We investigated the biomechanics of landing on smooth surfaces in crested geckos, *Correlophus ciliatus*, asking whether the incline of the landing platform alters impact forces and mid-air body movements. Using high-speed videography, we examined jumps from a horizontal take-off platform to horizontal, 45° and 90° landing platforms. Take-off velocity was greatest when geckos were jumping to a horizontal platform. Geckos did not modulate their body orientation in the air. Body curvature during landing, and landing duration, were greatest on the vertical platform. Together, these significantly reduced the impact force on the vertical platform. When landing on a smooth vertical surface, the geckos must engage the adhesive system to prevent slipping and falling. In contrast, landing on a horizontal surface requires no adhesion, but incurs high impact forces. Despite a lack of mid-air modulation, geckos appear robust to changing landing conditions.

## Introduction

Moving in an arboreal habitat often involves traversing an array of perches that vary in diameter, shape, incline, compliance, height from the ground, and roughness. Added to this is the network of branching patterns that constrain how and where an animal can move. These factors can alter the patterns of motion, but also locomotor performance^1–7^. Not surprisingly, many vertebrates exhibit adaptations for moving effectively in arboreal habitats, such as grasping limbs in mammals^8–10^ amphibians^10,11^, lizards^10,12,13^, and birds^10^, as well as using the body to grasp, as in snakes^14,15^. Other mechanisms of enhancing movement in an arboreal habitat include interlocking via claws^16^, wet adhesion^17^, and dry adhesion^18–20^. Most of these mechanisms have been studied under static conditions or during relatively steady locomotion^21–23^.

In addition to structural variation in the physical parts of the trees, arboreal habitats are inundated with gaps of various sizes, which are formed within and among plants. Although animals may simply avoid these gaps and move solely along the trunk and/or branches of a tree, many execute maneuvers to cross these gaps. There are several strategies for crossing gaps in fragmented habitats, such as reaching, swinging, gliding, and leaping^24–28^. The latter is often used for escaping predators^5,29–31^. In order to execute a successful leap, the animal must gain purchase on the take-off substrate and land without being injured. Much attention has focused on take-off biomechanics^31–33^, but fewer studies have examined landing among arboreal vertebrates^34–36^.

Both take-off and landing dynamics have been studied in anurans. When landing on a flat surface, anurans often make contact with their forelimbs first, indicating that they play an important role in stability and support during this phase of the jump^37,38^. As hop distance increases, pre-landing elbow extension increases and subsequent post-impact elbow flexion also increases^39^. However, the basal-most living frog family (Leiopelmatidae) extend their hindlimbs in addition to their forelimbs, thereby distributing the landing force across a range of ventral elements^40^. This is thought to reflect the more primordial way of jumping, which had developed at a time when frogs mainly jumped into water. Additionally, one study examined how an arboreal species of frog (*Trachycephalus resinifictrix*) lands on narrow arboreal perches^34^. They found that the adhesive ability is very important for hanging on, when a limb makes first contact. Unlike most anurans, landing in arboreal *Anolis* lizards, at least on a horizontal surface, typically begins with the contact of the hindlimbs, followed by the forelimbs^35,41^.

Geckos are well known for their complex hierarchical adhesive apparatus, and it was originally thought that the system was overbuilt, with safety factors exceeding 100^20,42^. These assertions were based on adhesive measurements under ideal conditions on artificial substrates. In reality, geckos are confronted with many different substrate characteristics, such as varying compliance, roughness, cleanliness, and wetness^43,44^. On rough surfaces, the available contact area may be very low^45,46^, decreasing safety factor considerably. Many geckos also live high in the canopy of forests^47–49^, and they often jump or fall from trees and land on either leaves or relatively smooth tree trunks^49,50^. This suggests that impact forces are likely quite high when this descent is arrested. Indeed, recent work has highlighted the fact that, when landing on leaves following a fall, adhesive forces may be exceedingly high^30^. Geckos are unique in that they often adopt a ‘sky diving’ posture, even in those geckos that lack an adhesive system^51^. This suggests that geckos will likely land with all four limbs ready to make contact upon collision. A recent study found that average landing angles (on a horizontal surface) in two species of arboreal gecko ranged from 30 to 40°^36^. A key question is whether geckos can re-orient themselves when landing on surfaces of different orientation in order to quickly engage the adhesive apparatus. Additionally, it is unclear if arboreal geckos are capable of landing on smooth vertical surfaces without falling. However, landing dynamics among geckos is poorly understood.

Using crested geckos (*Correlophus ciliatus*)*,* a species that commonly jumps in its natural habitat (A. M. Bauer, personal communication), we examined the role of landing surface incline on landing and mid-air dynamics. Specifically, we addressed the following three questions: (1) Does the incline of the landing surface alter impact forces? Given the differences in gravitational forces, we predict that impact forces would be lowest on the vertical surface. (2) Do geckos modulate their in-air body orientation in response to changes in landing surface incline? We predict that geckos will orient the long axis of their body parallel to the landing surface in order to ensure contact with all four feet shortly after impact. (3) Can geckos ameliorate the negative impacts of high impact forces? We predict that geckos will reduce the impact of landing by flexing their legs immediately following impact with the landing surface, as is common in frogs and anoles.

## Methods

### Animals

Four male *Correlophus ciliatus* were obtained from the pet trade and were held separately in terraria at the Botanic Garden of the University of Freiburg in Germany. All methods were carried out in accordance with relevant guidelines and regulations. Experimental protocols were approved by the Institutional Animal Care and Use Committee at the University of California, Riverside (AUP 20170039 issued to T.E.H.). For experiments, geckos were transported to the laboratory in plastic transport boxes. Body mass ranged from 35.9 to 49.2 g and was measured once before and once after the experimental period (no change was observed). Snout vent length (SVL) ranged from 11.37 to 12.74 cm.

### Experiments

In the experiments, a horizontal smooth glass plate (17.9 × 30.3 cm^2^) was used as the take-off platform. A wooden platform with a plastic-coated paper surface (21.0 × 29.7 cm^2^), which could be altered in height and angle, was used as landing platform with inclines of 0° (horizontal), 45° (hereafter termed “inclined”) and 90° (hereafter termed “vertical”). Both plates were fixed on a short side by a clamp attached to a pole. The surface for the landing platform was very smooth and maximized adhesion. Its black color was chosen to provide good visual contrast. Before each jump both surfaces were cleaned with ethanol.

**Figure 1:**
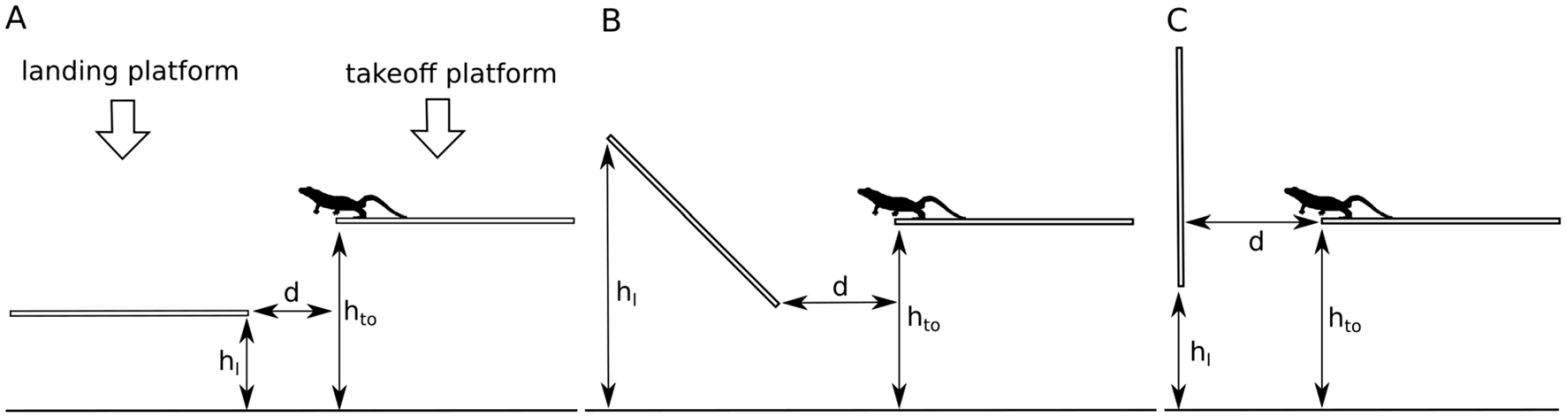
Setup for jumping experiments with landing platforms of different inclines. (**A**) A gecko jumping onto a lower horizontal platform. (**B**) A gecko jumping onto an inclined platform (45°). (**C**) A gecko jumping onto a vertical platform. Drawings are not scaled. Constructions that hold the take-off and landing platform are not shown. d: distance between platforms; h_l_: height of the landing platform; h_to_: height of the take-off platform.

After testing the willingness and ability of geckos to jump to platforms of different distances and heights, the following setup was used for the height of the take-off platform *h*_*to*_, the height of the landing platform *h*_*l*_ and the horizontal distance *d* (Fig. 1). For the horizontal setup (Fig. 1A) *h*_*to*_ was 52.0 cm, *h*_*l*_ was 26.0 cm and *d* was 43.5 cm. For gecko 1, a smaller distance *d* had to be used because of its lack of willingness or ability to jump farther. Therefore, *d* was 30.5 cm for the horizontal setup of gecko 1. It was assumed that differing platform distances would not impact the results because all geckos displayed different jump distances (i.e. they could land on any part of the platform). We were not incorporating jump distance in our study, so this should not impact our results. For the inclined setup (Fig. 1B), *h*_*to*_ was 52.0 cm, *h*_*l*_ was 58.3 cm and *d* was 23.5 cm. Finally, for the vertical landing platform (Fig. 1C), the distances were 52.8 cm for *h*_*to*_, 42.8 cm for *h*_*l*_ and 25.5 cm for *d*.

To record the jumps, a high-speed camera (MotionXtra NX4-S1 Camera, Integrated Design Tools, Inc., Tallahassee, Florida, USA) was used in combination with a 50 mm objective (Zeiss Planar T* 1.4/50, Carl Zeiss AG, Oberkochen, Germany) that was mounted on the camera via an adapter (F-/C-Mount-Adapter, Imaging Solutions GmbH, Eningen, Germany). The two vitrified walls in the working room created bright illumination conditions but the setup was additionally illuminated by a cold light source (Constellation 120 high-performance LED light source, Integrated Design Tools, Inc., Tallahassee, Florida, USA). The camera and the light source were both attached to a flexible stand (Manfrotto Magic Arm 244RC, Manfrotto, Cassola, Italy). The camera was oriented such that we obtained a lateral view of the take-off and landing surfaces (see supplementary video 1). The trials were recorded with IDT Motion Studio (Version 2.13.00, Integrated Design Tools, Inc., Tallahassee, Florida, USA) at 2000 frames per second (fps) and 300 μs exposure.

We obtained three trials per individual and treatment, with the following criteria being used: geckos must have been in the field of view during the entire duration of the trial, and individuals must not have slipped during take-off. Before the jumps the geckos were warmed to an appropriate operating temperature, which was monitored using a surface IR thermometer. Body temperatures were 31.3 ± 1.1 °C.

**Figure 2:**
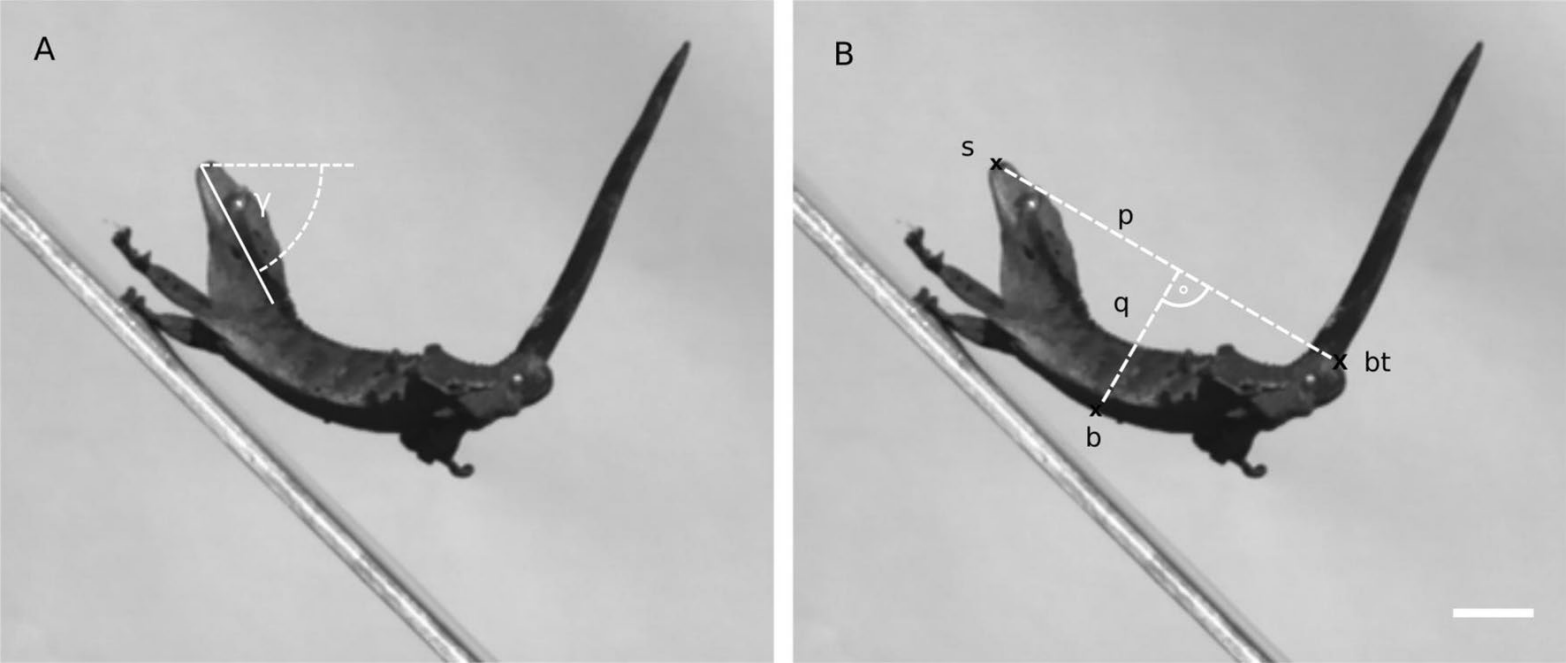
Video analysis of the landing process. (**A**) The landing angle γ describes the head posture at first surface contact and is measured between the horizontal line (dashed line) and the line passing through the snout and ventral part of the eye. (**B**) Three points tracked through the landing sequence: The tip of the snout (s), the belly (b) and the ventral base of the tail (bt). The distances of the perpendicular lines q and p were used to measure body curvature (see text). Scale bar represents 1 cm.

Take-off velocity was quantified at the frame of last contact with the substrate (see below for details of velocity calculation). Additionally, take-off angle was quantified in the same way as landing angle (see below and Fig. 2A), which was angle between the horizontal plane and the line between the tip of the snout and the ventral part of the eye. Rotation was quantified throughout the aerial phase of the jump.

To assess landing dynamics, we started digitizing 10 frames before first contact of any body part with the landing surface and stopped with the end of the landing process. This was defined as the frame after movement towards the platform ended. The time point of first contact, regardless of which part of the gecko made first contact, was set as *t* = 0 s.

Three points were digitized (Fig. 2B) representing the anterior, middle and posterior area of the gecko’s body: The most protruding point of the snout (s), a ventral point of the belly (b), which was estimated as the midpoint between the snout and ventral base of the tail (bt), and the ventral base of the tail. The coordinates of the three points were determined for each frame and used for further analysis. Body curvature was defined as the ratio of body deflection *q* and the linear distance *p* between snout and base of the tail (Fig. 2B):

1 $${\text{curvature}} = \frac{q}{p}$$

Maximum instantaneous body curvature was quantified. Finally, first contact of the gecko with the landing surface was categorized for each trial into “snout”, “forelimb”, “belly” and “hindlimb”, and was counted for each category and surface incline. The landing angle was measured as the angle between the horizontal plane and the line between the tip of the snout and the ventral part of the eye (Fig. 2A) at the moment of first contact, analogous to the determination of the take-off angle. A value of 0° indicates that the head is aligned with the horizontal plane. Landing duration was measured as the time between first contact with the surface and when the body reached its closest position to the substrate.

### Velocity and acceleration

The coordinates of the three body parts “snout”, “belly”, and “base of the tail”, were used to calculate the velocity *v* for each frame*:*

2 $$v= \frac{s}{\Delta t}$$

where *Δt* is time period between consecutive frames. The distance *s* corresponds to the displacement of the tracked body part:

3 $$s = \sqrt{{({x}_{2}- {x}_{1})}^{2}+({{y}_{2}- {y}_{1})}^{2}}$$

For each frame, acceleration *a* was calculated using the velocity differences between two frames:

4 $$a = \frac{{v_{2} - v_{1} }}{\Delta t}$$

In our study, deceleration indicates negative values of the measured acceleration. Therefore, maximal deceleration was determined for each trial by multiplying the most negative acceleration value by − 1.

### Impact force

**Figure 3:**
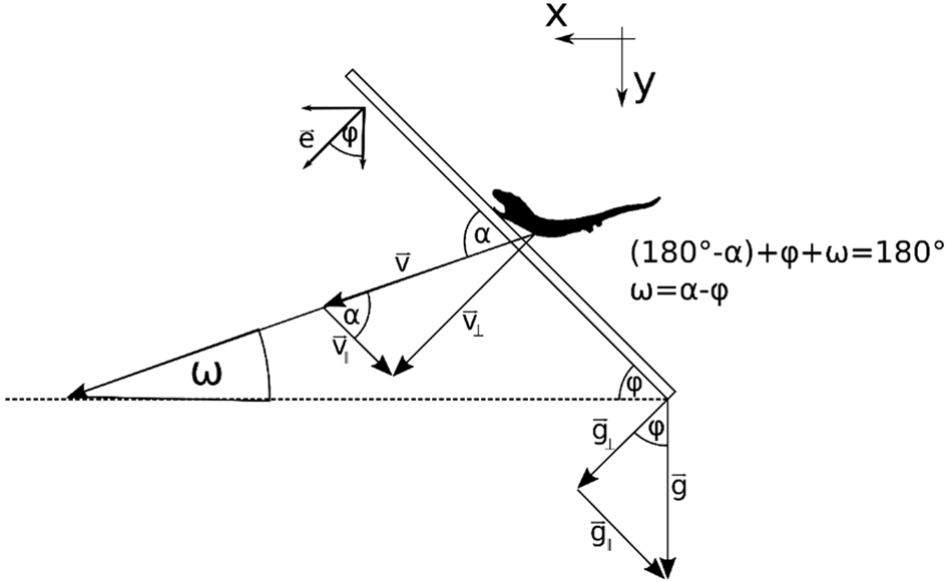
Factors determining the impact force. The velocity *v* of the gecko reaching the landing platform can be separated into velocity *v*_┴_ perpendicular to the surface and velocity *v*_*║*_ parallel to the surface. The perpendicular acceleration *g*_┴_ and the parallel acceleration *g*_*║*_ are part of gravitational acceleration *g* acting on the gecko’s body. The angle *α* lies between $\vec{v}$ and the platform. The angle *φ* corresponds with the platform incline towards the horizontal (dashed line).

The impact force *F*_*i*_ was calculated for the belly (approximate location of center of mass) using the following derivation. We start with the force vector:

5 $$\mathord{\buildrel{\lower3pt\hbox{$\scriptscriptstyle\rightharpoonup$}} \over F}=\left(\begin{array}{l}{F}_{x}\\ {F}_{y}\end{array}\right)=\left(\begin{array}{c}m \, {a}_{x}\\ m \, {a}_{y}+m\,g\end{array}\right)=\left(\begin{array}{c}m\,\frac{v}{T}\,{\cos}(\omega) \\ m\,\frac{v}{T}\,{\sin}(\omega) +m\,g\end{array}\right)$$

where *m* = mass of the body, *g* = gravitational acceleration, *a* = deceleration during landing, $v$ is the velocity, $T$ is the landing duration, and angles are outlined in Fig. 3. This force vector represents a general deceleration force. Since we oriented the coordinate system according to Fig. 3, the gravitational force pointing towards the ground has a positive sign.

The impact force acting on the gecko is perpendicular to the surface. Some intuition is gained by studying limiting cases: If the surface is aligned along the vertical direction (y-axis), the gravitational force acting in the y-direction would not contribute to the impact force and only the horizontal component of the force would matter. In contrast to this situation, the horizontal deceleration force would not contribute to the impact force if the surface is parallel to the ground. Thus, we projected the general expression of the impact force on a unit vector perpendicular to the surface:

6 $$\mathord{\buildrel{\lower3pt\hbox{$\scriptscriptstyle\rightharpoonup$}} \over e}=\left(\begin{array}{l} {\sin}(\varphi) \\ {\cos}(\varphi) \end{array}\right)$$

In the following, we derive how the impact force projected on the unit vector $$\mathord{\buildrel{\lower3pt\hbox{$\scriptscriptstyle\rightharpoonup$}} \over e}$$ leads to the final equation below. We start from the dot product between the two vectors:

7 $${\overrightarrow{F}\cdot \overrightarrow{e}=m\left\{\frac{v}{T} \, {\cos} (\omega)\, {\sin}(\varphi) +\left[\frac{v}{T} \, {\sin}(\omega) +g\right]{\cos}(\varphi) \right\}}$$

Using the angular relation $\omega =\alpha -\varphi$, given in Fig. 3, the equation becomes:

8 $${\overrightarrow{F}\cdot \overrightarrow{e}=m\,\frac{v}{T}\,\left[{\cos}(\alpha)\, {\cos}(\varphi)\, {\sin}(\varphi) +{\sin}(\varphi)\, {\sin}(\alpha)\, {\sin}(\varphi) +{\sin}(\alpha)\, {\cos}(\varphi)\, {\cos}(\varphi) - {\sin}(\varphi)\, {\cos}(\alpha)\, {\cos}(\varphi) \right]+m\,g\,{\cos}(\varphi)}$$

This yields the final equation for impact force:

9 $$F_{i} = m\, \frac{v}{T}\, {\sin}(\alpha) + m\,g\, {\cos}(\varphi) $$

The velocity perpendicular to the landing surface was calculated using the angle *α* between velocity vector $\vec{v}$ and the landing surface (Fig. 3):

10 $${v}_{\perp }= v\, {\sin}(\alpha )$$

The velocity vector of *v* was determined from the belly coordinates one frame before and directly at first contact with the landing surface:

11 $$\vec{v} = \left( \begin{gathered} \Delta x \hfill \\ \Delta y \hfill \\ 0 \hfill \\ \end{gathered} \right) = \left( \begin{gathered} x_{2} - x_{1} \hfill \\ y_{2} - y_{1} \hfill \\ 0 \hfill \\ \end{gathered} \right)$$

Δx and Δy were smoothed using a quintic spline algorithm (see below). Directional vectors of the platform were defined regarding the surface incline:

12 $$\vec{h}_{{0}^{\circ}} = \left( \begin{gathered} - 1 \hfill \\ 0 \hfill \\ 0 \hfill \\ \end{gathered} \right);\;\;\vec{h}_{{45}^{\circ}} = \left( \begin{gathered} - 1 \hfill \\ 1 \hfill \\ 0 \hfill \\ \end{gathered} \right);\;\;\vec{h}_{{90}^{\circ}} = \left( \begin{gathered} 0 \hfill \\ 1 \hfill \\ 0 \hfill \\ \end{gathered} \right)$$

Velocity vector $\vec{v}$ and directional vector of the landing surface $\vec{h}$ were used to calculate *α:*

13 $$\upalpha = \arccos \left( {\frac{{\vec{v} \cdot \vec{h}}}{{\left| {\vec{v}} \right| \left| {\vec{h}} \right|}}} \right)$$

### Statistics

For the calculations performed in this work Excel 2016 (Microsoft) was used. Statistical analysis was conducted with R (version 3.4.4^52^) using RStudio (version 1.1.442). Prior to data analyses, a quintic spline (custom code with the function *sm.spline*) was used to smooth the displacement data^53^. Incline of the landing platform was the categorial independent variable. We also included individual as a random factor, and all three trials for each individual were averaged prior to statistical analyses to avoid pseudoreplication. For the cases when the data met the assumptions of normal distribution and variance homogeneity, a repeated measures ANOVA (*lme* function) was used. This included the R nlme package (version 3.1-131.1^54^) and the multcomp package^55^. Normal distribution was tested using the Shapiro–Wilk test (*shapiro.test* function). For testing variance homogeneity, the Levene’s test (*leveneTest* function), including the car package (version 3.0-1^56^), was applied.

For the data that were not normally distributed or exhibited variance homogeneity, decadic logarithm was applied and the tests were rerun. In cases of non-parametric data, the data were analyzed using a Kruskal–Wallis test (*kruskal.test* function), including the R reshape package (version 0.8.7^57^). If there was no significant differences among individuals, they were removed from the model. If incline was found to be significant, the Dunn’s Test (*dunnTest* function) of the FSA package (version 0.8.20^58^) was chosen as a post-hoc test.

To determine if body size impacted landing velocity, landing duration, or landing angle, linear least squares regressions (using the *lm* function) with body mass were conducted. Since body size did not have any impacts, mass was not included in further tests. Body mass was already included in the quantification of impact force.

A p-value < 0.05 was considered significant, and all results are presented as mean ± standard error.

## Results

### General observations

**Figure 4:**
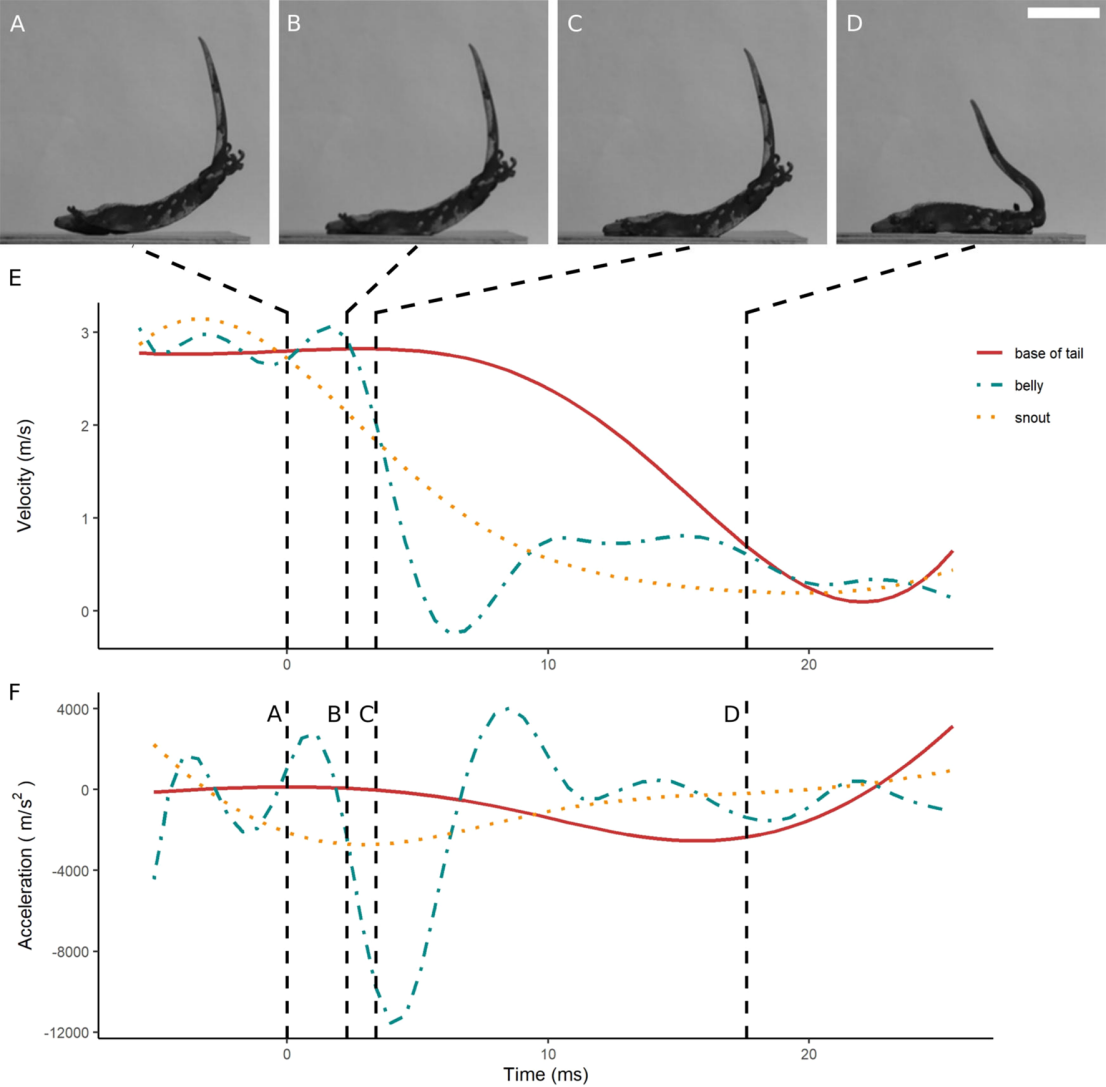
Representative landing process of a gecko jumping onto a horizontal platform. (**A**–**D**) Frames showing the belly (**A**), the snout (**B**), the forelimbs (**C**) and the hindlimbs (**D**) initiating contact with the landing platform. The time point of each frame is indicated on the plot by a vertical dashed line. (**E**) Velocity of snout (yellow dotted line), belly (blue dashed line) and base of the tail (solid red line) during landing. (**F**) Acceleration of snout, belly and base of the tail during landing. Scale bar represents 5 cm.

**Figure 5:**
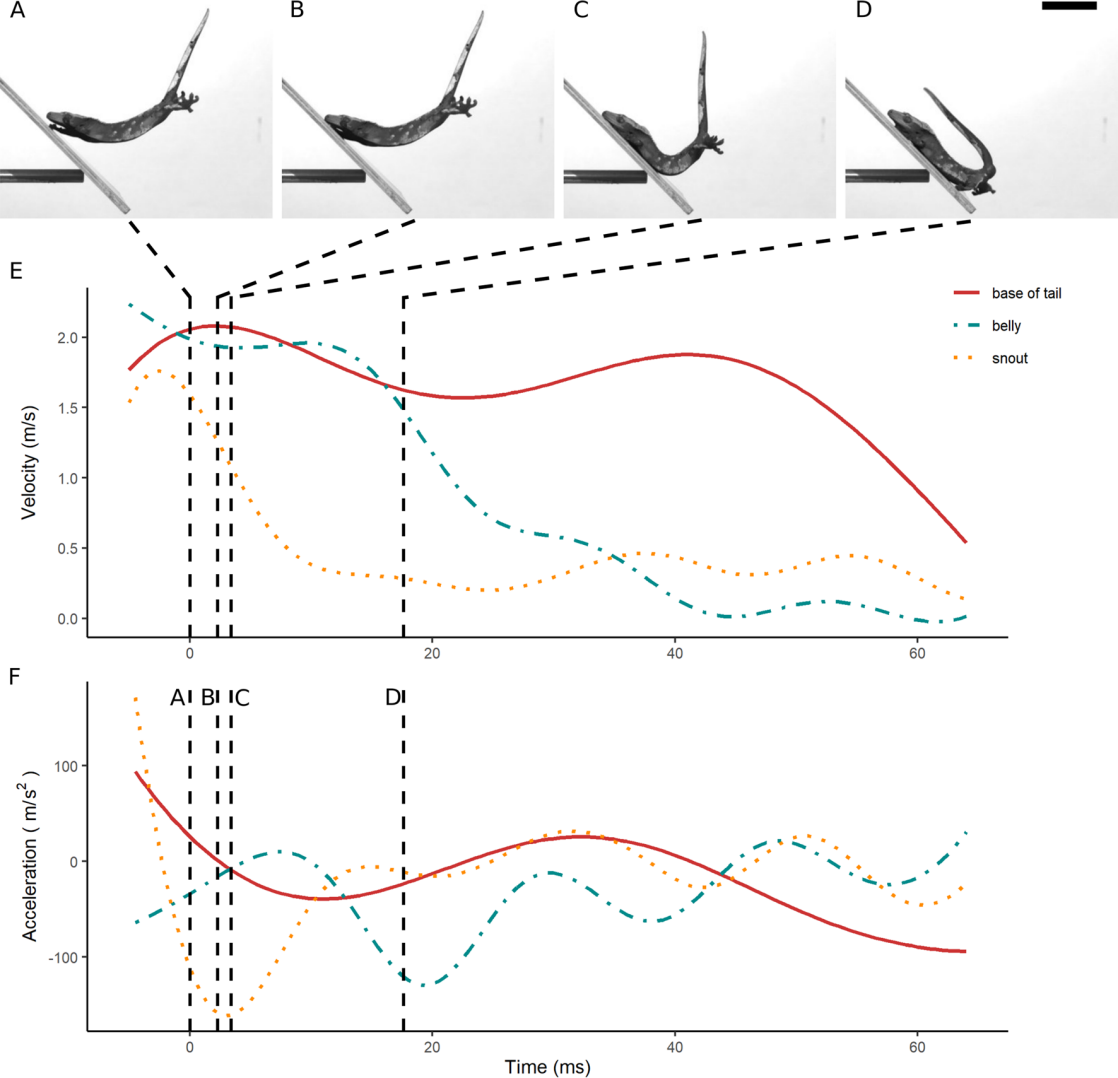
Representative landing process of a gecko jumping onto an inclined (45°) landing platform. (**A**–**D**) Frames showing the belly (**A**), the snout (**B**), the forelimbs (**C**) and the hindlimbs (**D**) initiating contact with the landing platform. The time point of each frame is indicated on the plot by a vertical dashed line. (**E**) Velocity of snout (yellow dotted line), belly (blue dashed line) and base of the tail (solid red line) during landing. (**F**) Acceleration of snout, belly and base of the tail during landing. Scale bar represents 5 cm.

**Figure 6:**
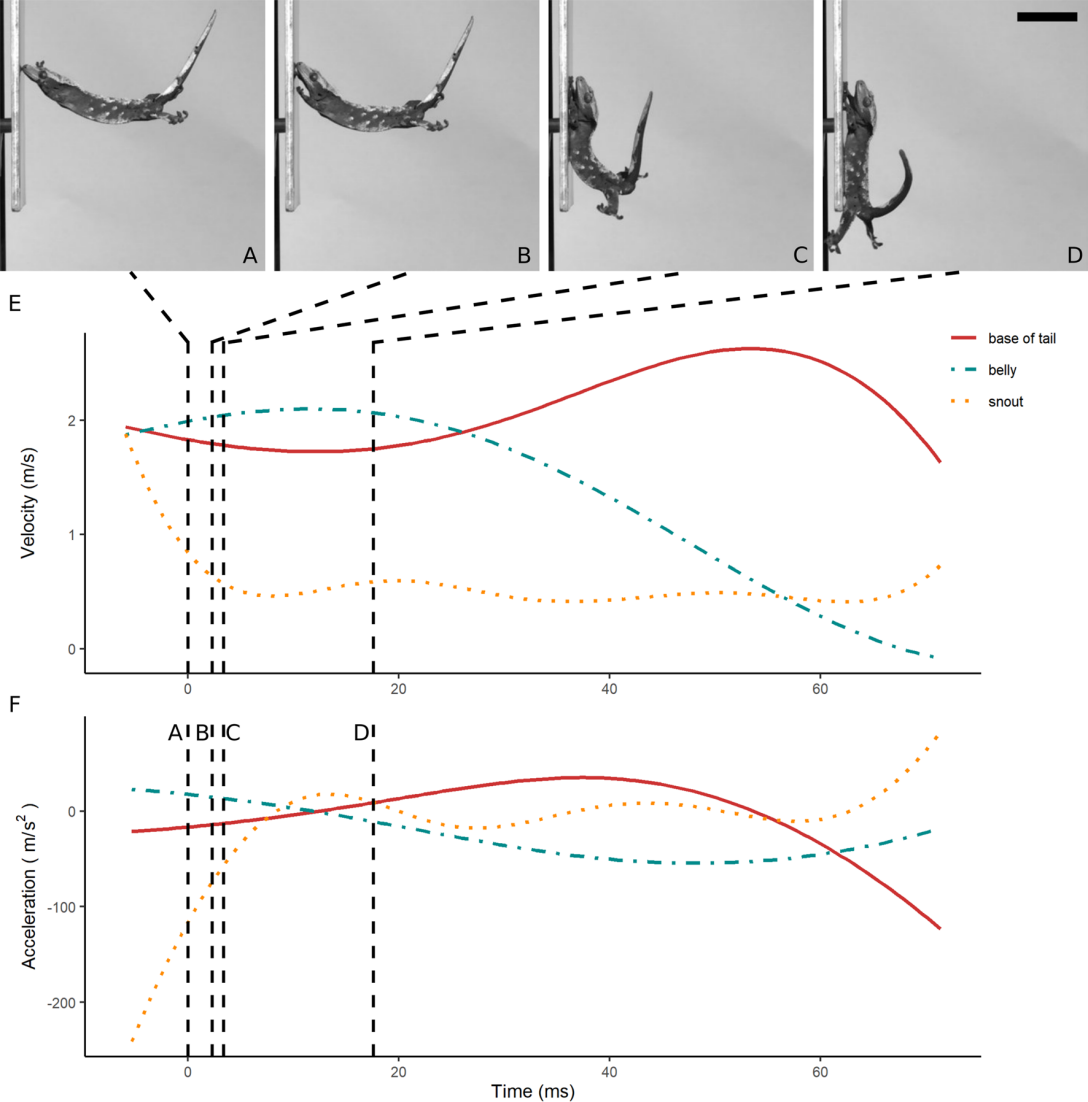
Representative landing process of a gecko jumping onto a vertical platform. (**A**–**D**) Frames showing the belly (**A**), the snout (**B**), the forelimbs (**C**) and the hindlimbs (**D**) initiating contact with the landing platform. The time point of each frame is indicated on the plot by a vertical dashed line. (**E**) Velocity of snout (yellow dotted line), belly (blue dashed line) and base of the tail (solid red line) during landing. (**F**) Acceleration of snout, belly and base of the tail during landing. Scale bar represents 5 cm.

Geckos in our study initiated take-off by fully extending their hindlimbs (supplementary video 1). Following the take-off, the geckos rapidly protracted their forelimbs and adopted a skydiving posture. Representative landing kinematic plots are shown in Figs. 4, 5, 6. The body part that contacted the landing surface first included the belly (Fig. 4A), the forelimbs (Fig. 5A), and the snout (Fig. 6A). Body curvature typically increased following contact (Figs. 4B,C, 5B,C, and 6B,C), eventually concluding the landing phase (Figs. 4D, 5D, and 6D). The body and the snout typically exhibited an initial decrease in velocity (Figs. 4E, 5E, and 6E), whereas the tail almost always kept moving after contact (e.g. Figure 5A–D). Acceleration during landing was more variable across treatments (Figs. 4F, 5F, and 6F).

### Take-off

Take-off velocity was significantly greater when the landing platform was horizontal (mean = 1.75 m s^−1^) compared to when it was either vertical (1.41 m s^−1^; p = 0.015) or inclined to 45° (1.44 m s^−1^; p = 0.025). Neither the angle of the body at take-off, nor the rotation during the aerial phase, were significantly impacted by the angle of the landing platform. There was modest positive correlation between the take-off angle and landing angle of the geckos (p = 0.029; r^2^ = 0.11).

### Point of first contact

**Figure 7:**
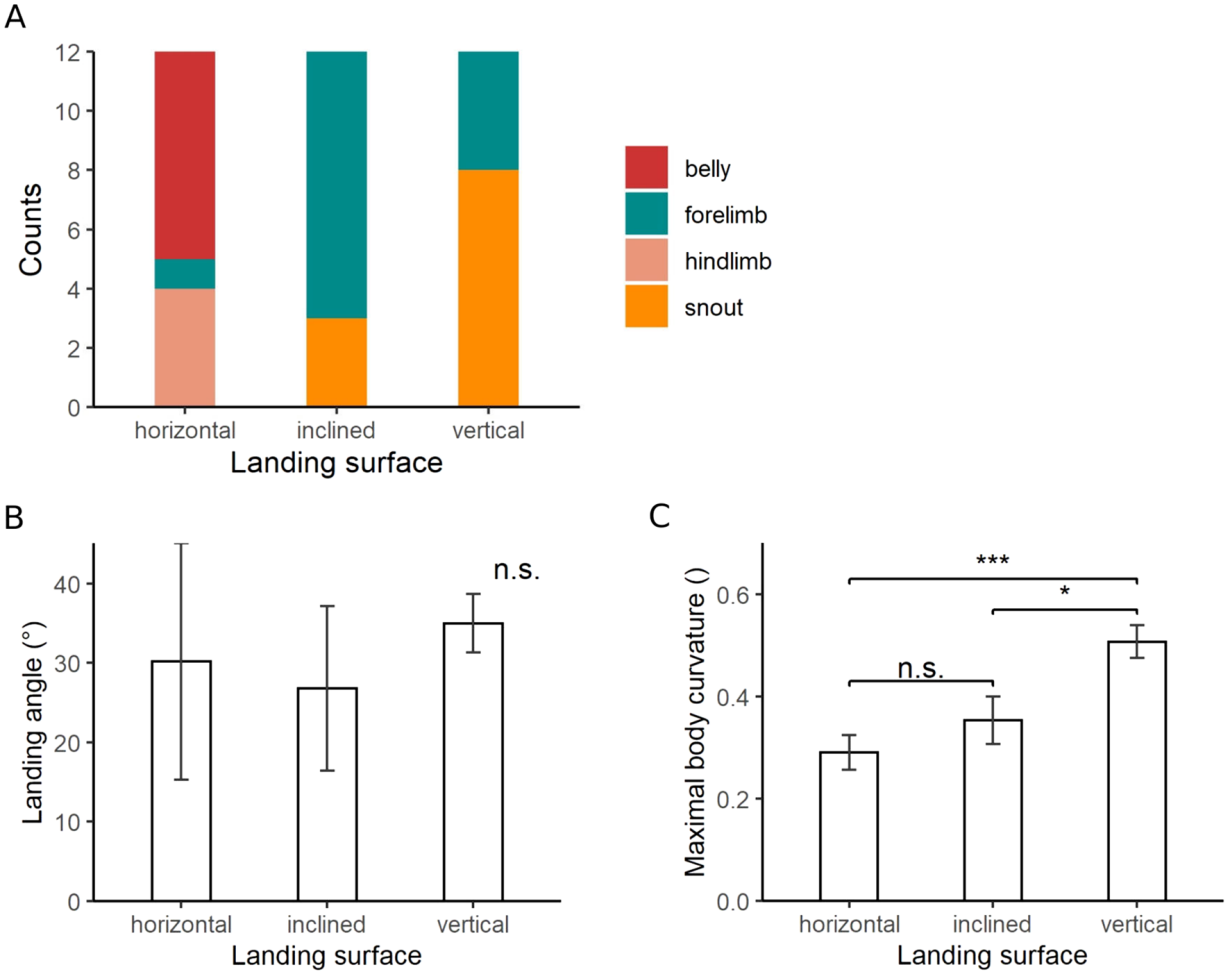
Body posture during landing. (**A**) Summary of which body part touched the landing platform first. (**B**) Landing angle, as measured in Fig. 2A, which is between the line intersecting the snout and eye line and the horizontal plane. (**C**) Maximum body curvature during landing, measured as the ratio of body deflection and the distance between snout and basis of the tail. Bar plots show mean values and standard errors. n.s.: no significance; *: 0.01 < p < 0.05; ***: p < 0.001.

On the horizontal landing platform, geckos made first contact with their belly and hindlimbs 58% and 33% of the time, respectively (Fig. 7A). Only the forelimb and snout made first contact on the inclined and vertical surfaces. On the inclined surface, the forelimb made first contact 75% of the time, whereas this occurred for 33% of the trials on the vertical treatment (Fig. 7A). The snout (67%) was the dominant site of first contact on the vertical treatment.

### Landing angle

The average value of the landing angle was 30.2 ± 8.2° for the horizontal platform, 26.8 ± 6.5° for the inclined platform and 35.0 ± 3.6° for the vertical treatment (Fig. 7B). After adding 3° to each angle, to avoid negative values, and applying decadic logarithm to gain variance homogeneity, a repeated measures ANOVA yielded an insignificant p-value of 0.52.

### Body curvature

Maximum curvature was 0.29 ± 0.02 for the horizontal, 0.35 ± 0.03 for the inclined, and 0.51 ± 0.02 for the vertical treatment (Fig. 7C). Applying a repeated measures ANOVA, the horizontal and vertical treatment were significantly different (p < 0.001), and the inclined and vertical treatment also differed (p = 0.027). Body curvature for the horizontal and inclined platform did not differ (p = 0.44).

### Landing velocity and deceleration

**Figure 8:**
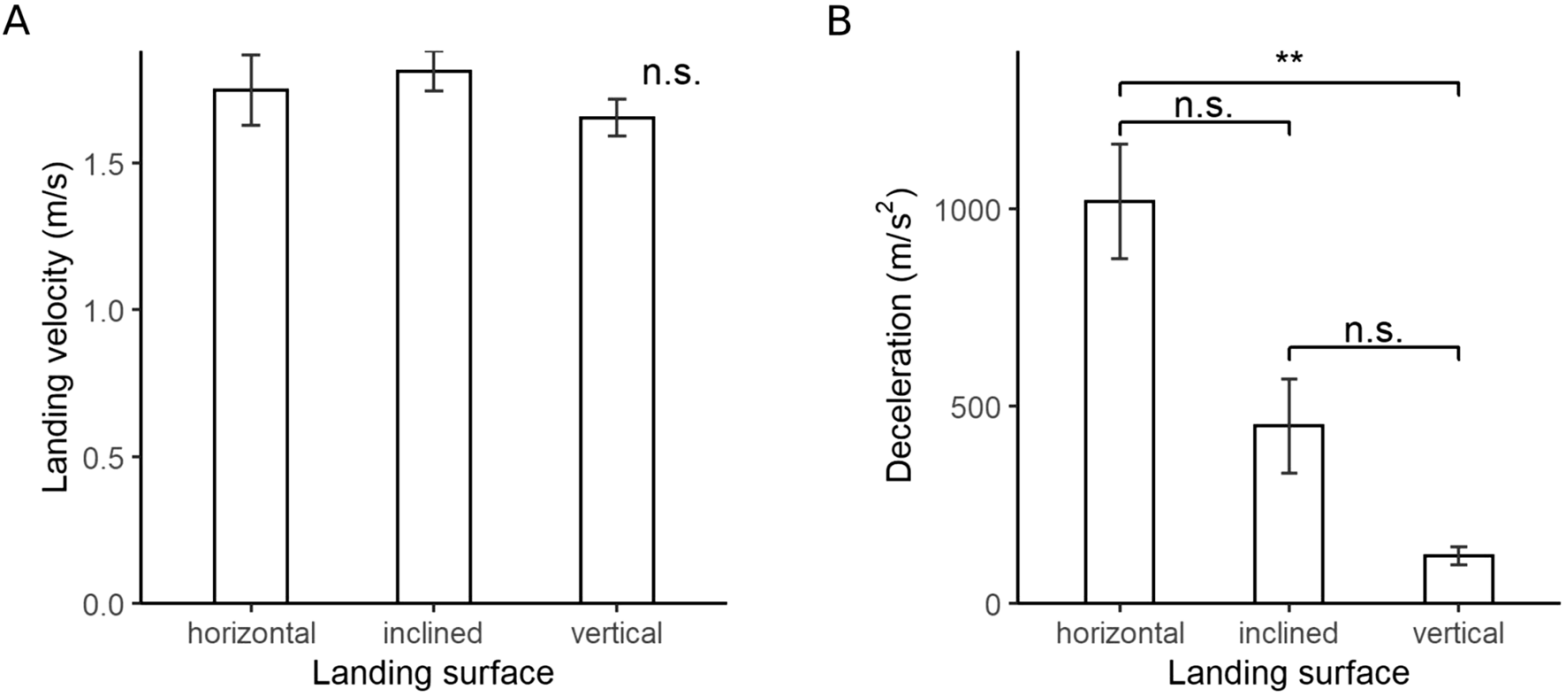
Landing kinematics. (**A**) Velocity of the belly perpendicular to the landing platform at the moment of first contact with the surface. (**B**) Maximum deceleration of the belly during landing. Bar plots show mean values and standard errors. n.s.: no significance; **: 0.01 < p < 0.001; ***: p < 0.001.

Landing velocity was 1.75 ± 0.12 m/s for the horizontal, 1.81 ± 0.07 m/s for the inclined and 1.66 ± 0.06 m/s for the vertical treatment (Fig. 8A). Since the velocity values did not show variance homogeneity even after log transformation (p < 0.05), a Kruskal–Wallis test was used. Neither individual (p = 0.62) nor incline (p = 0.39) significantly impacted landing velocity.

Peak landing deceleration decreased with platform incline (Fig. 8B). The average values of maximal deceleration were 1019 ± 72 m/s^2^ for the horizontal, 449 ± 59 m/s^2^ for the incline and 120 ± 11 m/s^2^ for the vertical platform (Fig. 8B). We again used a Kruskal–Wallis test due to the lack of variance homogeneity (p < 0.05). Peak deceleration was significantly different among inclines (p = 0.01), but not among individuals. The Dunn’s post-hoc test revealed a significant difference between the horizontal and the vertical incline (p = 0.007), but all other combinations were insignificant (Fig. 8B).

### Landing duration

**Figure 9:**
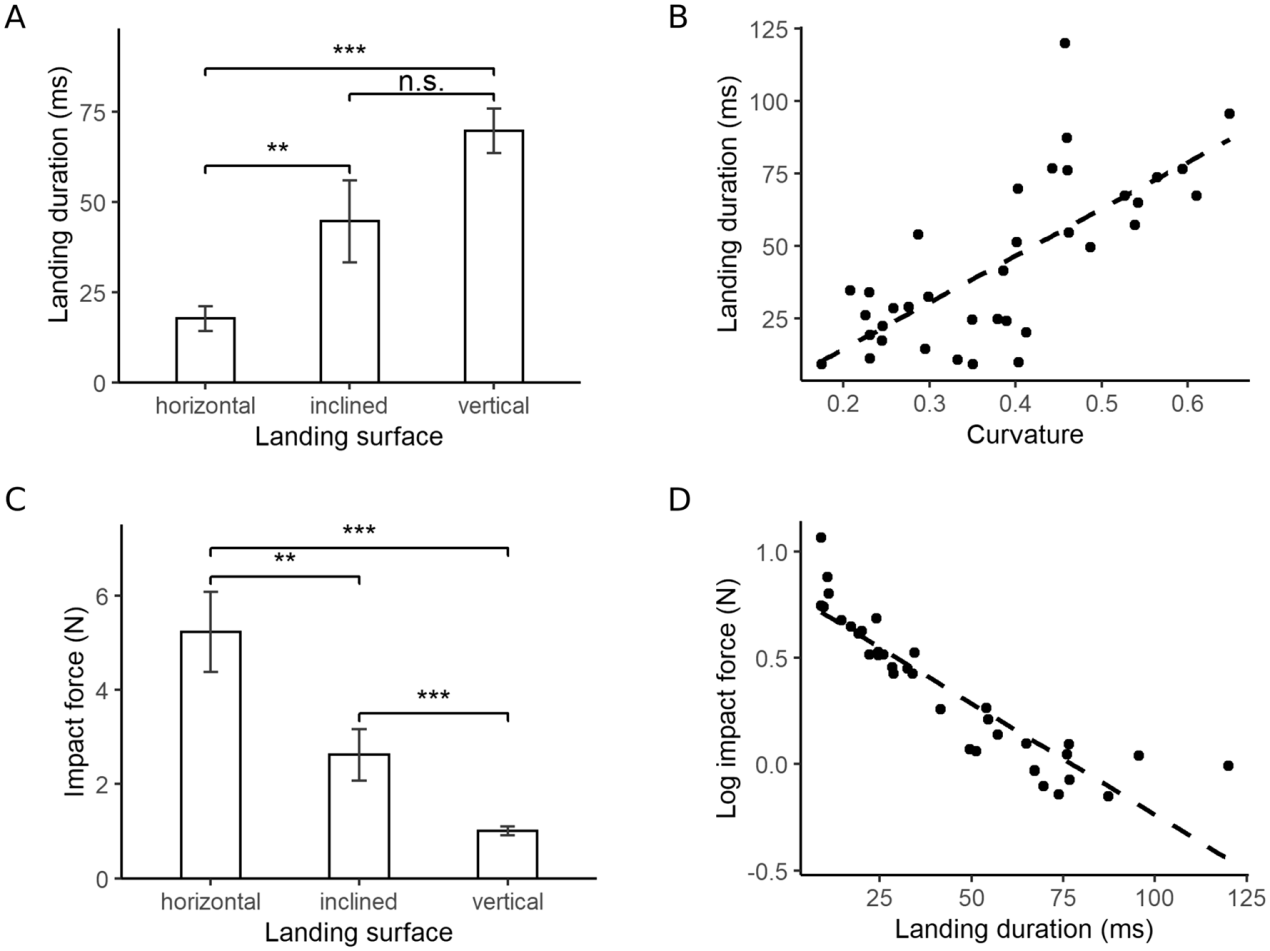
Influence of body curvature and landing duration on impact force. (**A**) Landing duration for each platform incline. (**B**) Correlation between maximal body curvature and landing duration (p = 5.5 × 10^–7^, r^2^ = 0.51). (**C**) Impact force acting on the gecko’s body during landing on the different surfaces. (**D**) Correlation between landing duration and log-transformed impact force (p = 4.3 × 10^–14^, r^2^ = 0.81). Bar plots show mean values and standard errors. n.s.: no significance; **: 0.001 < p < 0.01; ***: p < 0.001.

Landing duration was defined as the period between first contact and the moment the body reaches its closest point to the surface. The average landing durations for the horizontal, inclined, and vertical surfaces were 17.7 ± 3.4, 44.6 ± 11.4, and 69.7 ± 6.1 ms, respectively (Fig. 9A). After a log transformation, there was a significant difference between the horizontal and inclined treatments (p = 0.005; repeated measures ANOVA), as well as between the horizontal and vertical treatments (p < 0.001; repeated measures ANOVA). There was a positive correlation between body curvature and landing duration (p = 5.5 × 10^−⁠7^, r^2^ = 0.51; Fig. 9B).

### Impact force

Average impact force for the horizontal, inclined, and vertical treatments were 5.22 ± 0.85, 2.62 ± 0.55, and 1.01 ± 0.09 N, respectively (Fig. 9C). Using a repeated measures ANOVA on the log-transformed values, the horizontal and inclined treatments (p = 0.002), as well as the other combinations (p < 0.001) were found to be significantly different. There was a significant, and negative, correlation between landing duration and impact force (p = 4.4 × 10^−⁠14^, R^2^ = 0.81; Fig. 9D).

## Discussion

Crested geckos do not appear to actively modulate how they land when presented with platforms of different angles, suggesting that a single strategy is robust to changing conditions. The belly was often the first point of contact when landing on a horizontal platform, whereas the tip of the snout often made first contact when landing on a vertical surface. Thus, no significant differences in body angle were identified, as the geckos kept their head at an average angle between 26.8 ± 6.5° and 35.0 ± 3.6°. The positive values indicate that the snout was always angled up. There are important functional consequences for the strategy adopted by crested geckos, specifically involving impact force and the requirement for adhesion. Impact force is lower when a gecko jumps and lands on a vertically-oriented surface. However, this incurs the need to employ adhesion upon impact. Otherwise, the geckos risk slipping and falling to the ground. This biomechanical result now sets up an ecological question: do pad-bearing geckos prefer to jump to a vertical or horizontal surface in nature?

### Geckos reduce impact force passively through body curvature

The impact force experienced by a landing gecko can be quite high, especially when it jumps down from trees^30^. For *Thecadactylus rapicauda*, a species that lives in the forests of Central and South America^49^, impact forces were modeled to be as high as 17 N in extreme cases, potentially resulting in adhesive safety factors dipping below 1 (the point of failure)^30^. Given this, how might geckos reduce impact force, especially if they land sensitive body parts like their snout or belly? In our experiments, impact force was the highest for the horizontal surface (5.22 ± 0.85 N), whereas it was only 1.01 ± 0.09 N for the vertical surface. One reason for the differences is likely the contribution of gravitational force. It fully contributes to the impact on the horizontal surface, whereas it does not play a role for the vertical treatment. However, it is possible that the geckos are able to modulate other factors to reduce impact force.

Deceleration is the only component of the impact force equation that could be modulated. Maximal deceleration for the horizontal treatment was higher than for the vertical one. This was not due to greater velocities upon impact (Fig. 8A), but rather increased landing duration on the vertical platform (Fig. 9A). Duration was strongly, and negatively, correlated with impact force (Fig. 9D). How does a gecko modulate the duration of landing? The landing period begins with first contact with the landing surface and ends when the body is brought close to the platform. By maintaining body curvature when contact first begins (e.g. horizontal surface), there is a sequential impact of different parts of the body, typically starting with the belly (Fig. 7A). Had this not occurred, and the animal made contact with all parts simultaneously, then the impact forces would likely have been greater due to a shorter duration of impact.

The landing angle of the vertical surface, which resulted in an almost perpendicular collision (Fig. 6A), likely induced the curvature of the body. This allowed the body to exhibit a “reverse peeling” motion that also resulted in a delay in contact among body parts. This maximized the duration of impact. By doing so, this probably minimized the “bounce back” experienced by the gecko, allowing it to maximize frictional adhesion. It is important to note that a flexible body, permitting relatively high values of curvature (e.g. Figure 5C), is likely important for absorbing energy when landing. The sprawled posture of geckos likely precludes energy absorption via limb flexion. An alternative mechanism of minimizing the impact forces when jumping in an arboreal habitat is to land on relatively compliant perches, which are common in the wild^59^.

The lack of body angle modulation suggests that a single strategy works for most landing surfaces. This inherent flexibility points to a “one-to-many” mapping of posture to function. Without knowing what these geckos land on in nature, it is not possible to assign any ecological relevance to this flexibility. That said, it is unlikely that geckos in nature always land on surfaces with the same inclination.

### Adhesion and landing

A recent paper by Griffing et al.^60^ examined maximum digital adhesion force across a range of body sizes of *C. ciliatus* on the same substrate used in our study. Using their scaling equation (*Adhesion* = *1.041(body mass) – 0.69*), the crested geckos in our study could generate a maximum frictional adhesive force of approximately 10 N with a single forelimb. Forelimbs made first contact on the surface that was inclined to 45°, and impact forces averaged close to 2.5 N. Assuming that the forelimbs alone were bearing the weight, and that the downward component of the impact force (parallel to the platform) was substantial, the safety factors would be considerably lower than is typically thought for the gecko adhesive apparatus. Although the impact force is lower on the vertical surface, the gravitational force following impact will require the action of the adhesive system. It is possible that the hindlimbs could also engage following initial contact on an inclined or vertical surface, although their contact is delayed relative to the forelimbs (Figs. 5 and 6). Future work could include a force plate in the landing platform to examine the detailed three-dimensional forces during landing. Overall, our results suggest that geckos might preferentially land on vertical smooth surfaces when fleeing a predator to maximize safety factor, especially since this type of surface would be inaccessible to most vertebrate predators. Observations of these geckos jumping in nature is needed to elucidate the strategy adopted, as are studies incorporating rough landing surfaces.

### Tail function

The function of the tail during jumping has been studied in *Anolis* lizards, and it has been established that the tail contributes to the orientation of the body in the air. Loss of the tail, as in autotomy, changes the body angle but not the velocity or jumping distance^31^. Another recent study utilized *Agama* lizards and robots to determine how angular momentum perturbations were offset by the use of the tail during jumping^61^. In this case, a low-traction surface (from which the lizard jumped) induced a nose-down perturbation. This was counteracted by rapidly elevating the tail, which transferred momentum from the body to the tail to maintain body angle^61^. Finally, a recent study utilizing three-dimensional models and numerical simulations in gliding *Draco* lizards found that active tail movements enhanced gliding performance^62^. A previous study of crested gecko jumping found that the tail is elevated throughout the jump, thus altering the landing angle and sometimes even causing over-rotation upon landing^36^. We found a similar qualitative pattern, with the tail often being elevated throughout the jump (see Figs. 4, 5, and 6). Thus, crested geckos appear to naturally experience a nose-down tendency during jumping. Although unstudied, we would assume that a tailless crested gecko would experience a nose-down rotation, potentially causing a failed landing. This is potentially very important given that crested geckos do not regrow their tail following autotomy (personal observation). Although body angle was not calculated (nor any video obtained), a recent study found that jump success was not impacted by tail autotomy in Cape dwarf geckos (*Lygodactylus capensis*), even though they were landing on vertical surfaces^63^.

## Conclusions and future directions

We found that crested geckos do not modulate their in-air behavior when landing on surfaces of varying inclines. This did not alter their landing success, which means they are likely able to deal with different surfaces through passive mechanisms, such as bending of the body. Landing on vertical surfaces minimized impact forces by extending landing duration through body curvature. This potentially has large implications for how animals land on different arboreal surfaces following a fall or jump. Our results are a first step towards developing an ecomchanical model for landing in arboreal geckos^64^. Additional data are needed, including habitat use, fine-scale surface parameters, and performance on real-world surfaces.

The species used in our study is nocturnal, but due to filming constraints we obtained video in high-light conditions. It is unclear whether the results of our study would change if the light level is reduced, although recent work suggests that nocturnal geckos are not as sensitive to changes in light levels as diurnal geckos^65^. It is unclear when a crested gecko would jump in nature, but two likely scenarios are when foraging at night or when disturbed by a predator while it is hidden during the day. Field observations are needed to understand the ecological contexts of jumps.

Landing on compliant surfaces is a mechanism for minimizing impact forces^59^. We only used rigid landing platforms, but future studies should incorporate other ecologically-relevant landing surfaces. It might be expected, as suggested for primates by^66^, that geckos would jump from less compliant surfaces to minimize energy loss and land on compliant supports in order to absorb energy and minimize the impact force.

Finally, crested geckos are fairly large, and they may only jump to escape predation. This might explain the relatively stereotyped body position during the jump, in that they would likely jump down and land on cantilevered leaves (as in^30^). There are other geckos that are likely more agile jumpers, possibly utilizing this mode of locomotion when catching prey. Thus, they may be more likely to exhibit more advanced modulation of their body during jumping. Our approach should be extended to other species of gecko, such as those from the genus *Phelsuma*.

## Supplementary Information

Supplementary Legends.

Supplementary Video S1.

Supplementary Video S2.

Supplementary Video S3.

Supplementary Information.

## Data Availability

The data spreadsheet is included in the supplementary information.
