# Supplementary material for: Jumping with adhesion: landing surface incline alters impact force and body kinematics in crested geckos
Source: Sci Rep. 2021 Nov 29;11:23043. doi: 10.1038/s41598-021-02033-4 (PMC8630229; doi:10.1038/s41598-021-02033-4)
Supplement: Supplementary file 1 — Supplementary Legends. [file 41598_2021_2033_MOESM1_ESM.docx]

Supplemental videos:

Supplemental video 1: A video of a crested gecko landing on a horizontal smooth surface. The video was recorded at 2000 fps and played back at 20 fps.

Supplemental video 2: A video of a crested gecko landing on a inclined (45 degrees) smooth surface. The video was recorded at 2000 fps and played back at 20 fps.

Supplemental video 3: A video of a crested gecko landing on a vertical smooth surface. The video was recorded at 2000 fps and played back at 20 fps.
